# Cortical gene transcription response patterns to water maze training in aged mice

**DOI:** 10.1186/1471-2202-12-63

**Published:** 2011-06-29

**Authors:** Sung-Soo Park, Alexis M Stranahan, Wayne Chadwick, Yu Zhou, Liyun Wang, Bronwen Martin, Kevin G Becker, Stuart Maudsley

**Affiliations:** 1 Receptor Pharmacology Unit, National Institute on Aging, Baltimore, MD 21224 USA; 2 Physiology Department, Georgia Health Sciences University, 1120 15th St., Augusta, GA, 30912 USA; 3 Metabolism Unit, National Institute on Aging, Baltimore, MD 21224 USA; 4 Gene Expression and Genomics Unit, Research Resources Branch, National Institute on Aging, Baltimore, MD 21224 USA

## Abstract

**Background:**

The hippocampus mediates the acquisition of spatial memory, but the memory trace is eventually transferred to the cortex. We have investigated transcriptional activation of pathways related to cognitive function in the cortex of the aged mouse by analyzing gene expression following water maze training.

**Results:**

We identified genes that were differentially responsive in aged mice with accurate spatial performance during probe trials or repeated swimming sessions, relative to home cage conditions. Effective learners exhibited significantly greater activation of several pathways, such as the mitogen-activated protein kinase and insulin receptor signaling pathways, relative to swimmers. The genes encoding activity-related cytoskeletal protein (Arc) and brain-derived neurotrophic factor (BDNF) were upregulated in proficient learners, relative to swimmers and home cage controls, while the gene encoding Rho GTPase activating protein 32 (GRIT) was downregulated. We explored the regulation of Arc, BDNF, and GRIT expression in greater morphological detail using in situ hybridization. Recall during probe trials enhanced Arc expression across multiple cortical regions involved in the cognitive component of water maze learning, while BDNF expression was more homogeneously upregulated across cortical regions involved in the associational and sensorimotor aspects of water maze training. In contrast, levels of GRIT expression were uniformly reduced across all cortical regions examined.

**Conclusions:**

These results suggest that cortical gene transcription is responsive to learning in aged mice that exhibit behavioral proficiency, and support a distributed hypothesis of memory storage across multiple cortical compartments.

## Background

Aging is accompanied by changes in performance across a variety of cognitive domains. Numerous studies suggest that cognitive stimulation prevents declining neuronal function and enhances structural plasticity in the hippocampus. For example, training on hippocampus-dependent tasks has been shown to promote both adult neurogenesis [1] and increases in hippocampal dendritic spine density [2] in young animals. The underlying molecular mechanism for these enhancements likely involves changes in synaptic plasticity and neurotrophic factor expression. Brain-derived neurotrophic factor (BDNF), which promotes synaptic plasticity [3], also enhances hippocampal learning in adult animals [4,5]. Factors that enhance BDNF expression, such as exercise, also improve performance on hippocampus-dependent tasks in aged animals [6,7]. However, the transcriptional changes associated with cognition are less frequently investigated in central nervous system regions outside the hippocampus.

It may seem counterintuitive to look for mechanisms that support hippocampus-dependent memory outside of the hippocampus. However, cortical structures contribute to higher-order information processing and may contribute to some aspects of performance in the Morris water maze. For example, damage to the dorsolateral band of the entorhinal cortex impairs water maze performance [8]. Moreover, expression of a constitutively active form of calcium-calmodulin kinase II (CaMKII) in the entorhinal cortex also reduced spatial memory formation in the water maze [9]. Pharmacological disruption of extracellular signal-regulated kinase (ERK) activity in the entorhinal cortex negatively regulates hippocampus-dependent learning in the water maze [10], suggesting that molecular mechanisms associated with synaptic plasticity in the entorhinal cortex may contribute to water maze performance.

The relationship between cortical neuronal activity and water maze learning is not unique to the entorhinal cortex. Lesions of the retrosplenial cortex impair water maze performance [11,12]. Lesions to the visual and parietal cortices impair water maze learning, but pre-training with a visible platform abolished this relationship, suggesting that these cortical regions participate in water maze strategy learning, rather than spatial memory formation [13]. Based on the results of lesion studies [8], genetic manipulations [9], and pharmacological manipulations [10], it is likely that synaptic plasticity in the cortex, as well as the hippocampus, contributes to water maze learning.

The hippocampus is monosynaptically connected with multiple cortical areas, including the entorhinal and retrosplenial cortices [14,15]. These connections contribute to information processing and memory, and cortical brain regions are likely to show a variety of altered transcriptional profiles following cognitive stimulation. In this study, we used microarray and functional pathway analysis to reveal that goal-directed water maze learning induces transcripts associated with synaptic plasticity in the cortex of aged mice that perform effectively in the water maze. Proficient learning was accompanied by widespread changes in the expression of Arc, BDNF, and GRIT across multiple cortical regions involved in the cognitive and sensory aspects of water maze training. These observations suggest that, in a subset of aged mice that retain the capacity for efficient spatial learning, memory recall recruits transcriptional networks associated with cortical plasticity.

## Methods

### Animals and experimental design

Animal care and experimental procedures followed NIH guidelines and were approved by the National Institute on Aging Animal Care and Use Committee and all effort was made to minimize pain or discomfort. Male C57BL/6 mice at twenty months of age were randomly assigned to learning (n = 10), swimming (n = 6), or home cage (n = 6) groups. Home-cage control mice were sacrificed directly out of the cage, while learners received five days of training in the Morris water maze with a probe trial on the sixth day. For the learners, only mice that engaged in a goal-directed search were used for analysis and subsequent microarray. Mice that spent more than half of each trial floating (defined as moving at a speed of less than 2.0 cm per second) or engaging in thigmotaxis (defined as remaining within 4.0 cm of the perimeter of the pool) were not included in the study. Four mice met exclusion criteria in the learning group; six mice engaged in goal-directed learning based on these parameters. Swimmers received six days of swimming without a target in the water maze. Learners (n = 6), swimmers (n = 6), and home cage controls (n = 6) were sacrificed ninety minutes after the last learning or swimming trial. This time point was selected on the basis of previous experiments demonstrating sustained induction of cortical Arc mRNA expression following learning [16]. The mice were euthanized with Isoflurane, perfused with 0.9% saline, and subsequently the brain of each mouse was carefully removed from the skull. Half of the brain was dissected to remove the cortex for RNA extraction and microarray. The opposite hemi-brains were immersion-fixed in 4% paraformaldehyde in phosphate buffer for in situ hybridization.

All behavioral experiments involving learning and swimming were conducted in parallel by the same experimenter. Learners, swimmers, and home cage controls were processed together for all steps of the experiment, and all mice were aged in-house after being obtained from the same supplier (Jackson Laboratories, Bar Harbor, Maine, USA). We verified the health status of the mice by measuring serum glucose, 3-hydroxybutyrate, triglyceride, cholesterol, corticosterone, and insulin concentrations, and by visual inspection of the peritoneal cavity and brain to verify the absence of tumors. The results of the serum chemistry analyses have been reported previously [7].

### Water maze training

Water maze training took place as described previously [7]. Briefly, animals received five days of acquisition training, consisting of four trials per day, with an intertrial interval of approximately 10 min. Each trial lasted until the animal found the platform, or for a maximum of 60 seconds; animals that failed to find the platform within 60 seconds were guided there by the experimenter. On each trial mice were placed into the pool, facing the wall, with start locations varied across trials and days. One day after the last acquisition training session, animals were tested in a single 60 second probe trial without the platform. All training took place during the light phase, between 8:00 AM and 12:00 PM (lights on at 6:00 AM).

### RNA extraction and microarray

RNA extraction and microarray analysis were carried out as described previously [7]. RNA was isolated using the Qiagen RNeasy Mini Kit for animal tissues (Qiagen, Inc. Valencia CA). The RNA quality and quantity was checked using an Agilent 2100 bio-analyzer and the RNA 6000 nano-chips. Total RNA was used to generate biotin labeled cRNA using the Illumina TotalPrep RNA Amplification Kit (Ambion; Austin, TX, cat #IL1791). A total of 0.75 μg of biotin-labeled cRNA was hybridized at 58°C for 16 h to Illumina's Sentrix MouseRef-8 Expression Bead-Chips (Illumina, San Diego, CA). The arrays were washed, blocked and the labeled cRNA was detected by staining with streptavidin-Cy3. The arrays were scanned using an Illumina BeadStation 500 × Genetic Analysis Systems scanner and the image data extracted using the Illumina BeadStudio software, Version 3.0.

### Microarray data analysis

Microarray data were analyzed using DIANE 6.0, a spreadsheet-based microarray analysis program based on the SAS JMP7.0 system. Raw microarray data were subjected to filtering and Z normalization and tested for significant changes as described previously [7]. Briefly, sample quality was analyzed by scatter plot and gene sample Z-score based hierarchical clustering to exclude possible outliers. Initial filtering identified genes with Z-ratio ≥1.96, with the Z-ratio derived from the difference between the averages of the observed gene Z scores, divided by the standard deviation of all of the differences for that particular comparison. We were able to detect approximately 20,000 genes after filtering. Genes were then refined by calculating the false discovery rate (FDR), which controls for the expected proportion of falsely rejected hypotheses, and including only those genes with FDR <0.05. These data were further analyzed using a one-way ANOVA design with significance set at p < 0.05. The ANOVA design compared across experimental conditions (home cage, swimming, or learning). This allowed us to identify transcripts that differed in their intensity for learning and swimming animals.

### Bioinformatic analysis

After identifying individual genes that were significantly regulated by different experiences in learning and swimming mice, the gene lists were analyzed using functional annotational clustering, *i.e*. gene ontology (GO), signaling pathway analysis (using pathways defined by the Kyoto Encyclopedia of Genes and Genomes (KEGG) (http://www.genome.jp/kegg/) and functional gene network analysis (Ingenuity Pathway Analysis, http://www.ingenuity.com/). The GO and KEGG pathway analysis was performed using WebGestalt (http://bioinfo.vanderbilt.edu/webgestalt/), a web-based gene-set analysis toolkit as previously described by Martin et al. [17]. This application allows the comparison of the expression frequency of a specific gene in the experimental set to be compared to its expression frequency in a background murine gene set that is maintained at WebGestalt. Using the Network Explorer function in Ingenuity Pathway Analysis (IPA), the most significantly populated gene networks for the specific genesets described were calculated based on the percentage population (by network eligible focus molecules), mediated by the gene identities of the input data sets, of the resultant gene networks. A comprehensive overview of the use of network algorithms and significance generation is given by Calvano et al. [18]. The genes were only considered to be network eligible if they were known to interact with at least one other molecule in the network. In each case more than two genes were required to adequately populate a given network with at least a p value of less than 0.05.

### In situ hybridization

Hemibrains were sectioned on the coronal plane at 40 μm thickness in a 1:12 series on a freezing microtome. Sections were stored at 4°C in 4% paraformaldehyde in phosphate buffer. For Arc probe synthesis, we used a previously described plasmid [19] that contains nearly full-length cDNA (~3 kbp) of the Arc transcript. The plasmid was cut with EcoRI restriction enzyme (Promega) to expose the T7 binding site. Quality of the cut plasmid was verified by gel electrophoresis and the probe was phenol/chloroform extracted and precipitated in ethanol at -80°C. ^35^S-UTP labeled riboprobe was then generated using the Maxiscript kit (Ambion), followed by another round of phenol-chloroform extraction at -80°C. The final probe was resuspended in RNase-free water and the specific activity was determined by scintillation counter.

For BDNF and GRIT riboprobes, we generated probe templates as described previously [20]. For BDNF, right primer gtctgacgacgacatcactg, left primer ccatgggattacacttggtc; for GRIT, right primer acacttctgagccaagcaac, left primer agggagtggtaatcctccag. PCR products were verified by restriction endonuclease digestion, then amplified further with the same PCR primers that had been modified by the addition of T7 or SP6 RNA polymerase binding sites. PCR products containing T7 and SP6 extensions were purified by SVgel and a PCR cleanup kit (Promega). 35S-UTP labeled riboprobe was then generated using the Maxiscript kit (Ambion). Probes were extracted, resuspended, and counted using a scintillation counter as described above for the Arc plasmid.

In situ hybridization was carried out as described by Stranahan et al. [20]. All tissue for a single probe was processed in the same reaction. Free-floating tissue sections were washed in 0.75% glycine in 0.1 M phosphate buffer two times, followed by a single wash in phosphate buffer. Sections were then exposed to a Proteinase K buffer containing 1.0 μg/mL proteinase K for 30 minutes at 37°C. Sections were then treated with acetic anhydride solution (11.3% triethanolamine, 0.25% acetic anhydride, 0.04 M acetic acid) for 10 minutes at room temperature. This was followed by two 15-minute washes in 2× sodium chloride/citrate buffer (SSC buffer; 20× concentration, 3 M NaCl, 0.3 M sodium citrate). Next, sections were transferred to a hybridization buffer containing 20% formamide, 0.4× Denhardt's solution, 4% dextran sulfate, and 1.6× SSC) supplemented with 0.25 mg/mL tRNA, 0.33 mg/mL sheared salmon sperm DNA, 100 mM DTT, and 1 × 10^7 ^cpm/mL ^35^S-UTP-labeled probe for overnight reaction at 60°C. The following day, sections were washed at 60°C in 4xSSC/0.01M DTT and 2× SSC/50% formamide and then incubated with RNase (20 μg/mL) at 37°C for 30 min. Sections were washed with progressively decreasing concentrations of SSC before mounting on slides. Slides were dried overnight, exposed to a phosphorimager screen, and quantified by using ImageQuant (GE Healthcare). Digital images were acquired of a minimum of three anatomically matched sections in order to generate an average optical density measure for each animal. The region of interest was outlined by hand by a researcher who was blind to group conditions.

### Anatomical sampling

Cortical regions were identified based on the atlas of Paxinos and Franklin [21]. For the anterior cingulate cortex, we grouped cingulate cortex area 1 and cingulate cortex area 2 together with the cingulate/retrosplenial cortex region (Bregma +2.34 mm through Bregma -0.82 mm). To analyze the auditory cortex, we sampled from the ventral area of the secondary auditory cortex, the dorsal area of the secondary auditory cortex, and the primary auditory cortex (Bregma -1.70 mm through Bregma -3.64 mm). For the entorhinal cortex, we sampled from both the lateral and medial entorhinal areas (Bregma -2.18 mm through Bregma -5.02 mm). In the motor cortex, we combined the primary and secondary motor cortices (Bregma +2.46 mm to Bregma -1.32 mm). During analysis of the somatosensory cortex, we took sections containing primary somatosensory cortex and the secondary somatosensory cortex (Bregma +1.94 mm through Bregma -2.30 mm). When sampling from the retrosplenial cortex, we combined the retrosplenial agranular cortex and the retrosplenial granular cortex (Bregma -0.94 mm through Bregma -4.96 mm). Lastly, when measuring gene expression in the visual cortex, we sampled from the primary visual cortex and the lateral and medial areas of the secondary visual cortex (including both the mediomedial and mediolateral subdivisions); sampling of the visual cortex extended from Bregma -1.94 mm to Bregma -5.02 mm.

### Statistics

In situ hybridization data were analyzed using one-way ANOVA followed by Bonferroni post hoc testing to compare gene expression within a specified cortical region across learners, swimmers, and home cage controls. To compare gene expression across cortical regions implicated in the cognitive and sensorimotor aspects of water maze learning, we used a 2 × 3 ANOVA design with Bonferroni's post hoc test. Behavior data were compared between learners and swimmers using one-way repeated measures ANOVA followed by Bonferroni's post hoc. For all analyses, statistical significance was set at p < 0.05.

## Results

### Spatial memory performance in aged mice

For the assessment of both goal-directed and non-goal-directed activity, we employed the Morris water maze with either no escape platform (non-goal-directed; swimming) or with the standard hidden escape platform (goal-directed; learning). Aged mice in the learning group were included or excluded based on behavioral criteria as described in Materials and Methods and only proficient learners were used for the microarray analysis. In this respect, we compared aged mice that experienced effective learning, aged mice that engaged in physical activity, and aged home cage control mice.

**Figure 1:**
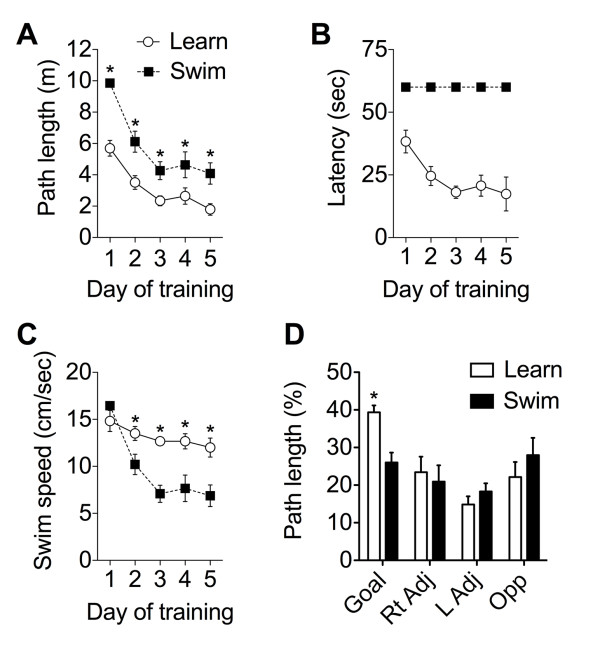
**Behavioral performance of learners and swimmers in the water maze**. (**A**), Both swimmers and learners decreased their swimming distances with repeated exposure to the maze. (**B**), While learners found the platform more quickly with repeated training, swimmers were exposed to one minute of swimming without a platform in the maze. (**C**), Swim speed was fairly constant for mice that learned to find the hidden platform, but decreased for mice that swam without a platform in the maze. (**D**), Mice trained to find the hidden platform exhibit a spatial bias for the target location during the probe trial, while mice that swam without a platform do not show spatial bias. Abbreviations: Rt Adj, right adjacent quadrant; L Adj, left adjacent quadrant; Opp, opposite quadrant.

Both the swimming group and the selected group of proficient learners reduced their path lengths over the course of training, with effective learners maintaining consistently shorter path lengths than swimmers (Figure 1A; F_1,10 _= 22.27, p = 0.008). The selected group of proficient learners also escaped to the platform more quickly with repeated training (Figure 1B), while swimmers had no opportunity to escape and swam for the full sixty seconds of the trial. Swimmers decreased their swim speeds over five days of training, while the subset of effective learners did not (Figure 1C; F_1,10 _= 12.45, p = 0.005). The learner mice, despite their advanced age, demonstrated an ability to learn the task as evidenced by their spatial bias for the goal quadrant during the probe trial on the sixth day (Figure 1D; F_1,39 _= 3.26, p = 0.03). Swimmers exhibit no spatial bias.

### Learning or swimming differentially regulate cortical transcription in aged C57Bl6 mice

**Figure 2:**
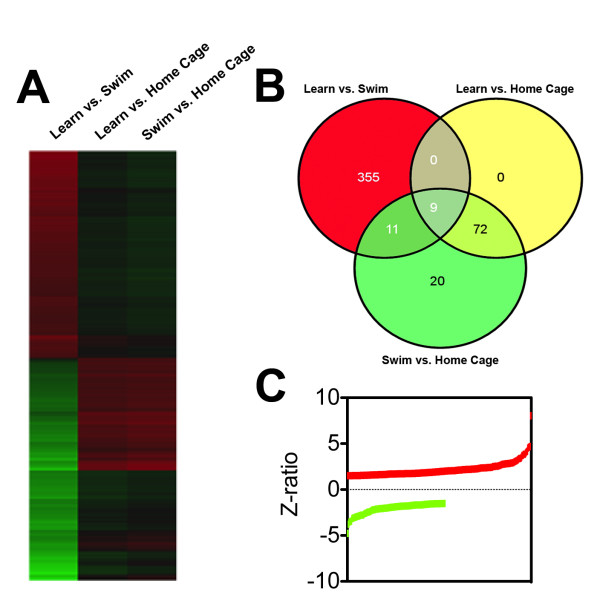
**Learning and swimming recruit distinct gene expression profiles**. (**A**), Genetic heatmap plot showing the cortical genes regulated, compared to home cage sedentary animals, by learning or swimming protocols. Red represents upregulation, while green shows downregulation. (**B**), Venn diagram showing the specific transcripts expressed in the aged mouse cortex following learning and swimming protocols. Learning-induced transcripts represent a subset of genes expressed following learning and swimming. This suggests that cortical gene expression partitions learning-related genes within physical activation- and arousal-related genes. (**C**), Data plot of individual gene z ratios of the learning versus swimming protocol analysis. Z ratios indicating both directions of relative gene regulation were observed.

To compare the effects of swimming and learning on cortical gene transcription, we identified transcripts that showed significantly different expression levels between learners and swimmers, compared to the sedentary home-caged animals (Figure 2A). Learners showed 355 gene transcripts whose expression levels were significantly different from swimmers (Figure 2B; for a full list of transcripts that distinguish between learning and swimming, see Additional File 1). Among the upregulated genes, Arc (activity regulated cytoskeletal-associated protein) showed the largest increase in learners relative to swimmers. Additional learning-specific transcripts include dual specificity phosphatases (Dusp1, GenBank ID:19252; Dusp6, GenBank ID:67603), Jun-B oncogene (Junb, GenBank ID:16477), synaptotagmin 13 (syt13, GenBank ID: 80976), and cytochrome b5 reductase 4 (cyb5r4, GenBank ID: 266690). Interestingly, the expression of brain derived neurotrophic factor (Bdnf, GenBank ID:12064) and inducible nerve growth factor (Vgf, GenBank ID: 381677) was stronger in learners than in swimmers, implying that induction of growth factor expression occurs more following learning than swimming. Approximately two-thirds of the transcripts that were responsive to effective learning were upregulated, as indicated by positive Z-scores as shown (Figure 2C).

### Differential signaling pathway activation following learning and swimming

**Figure 3:**
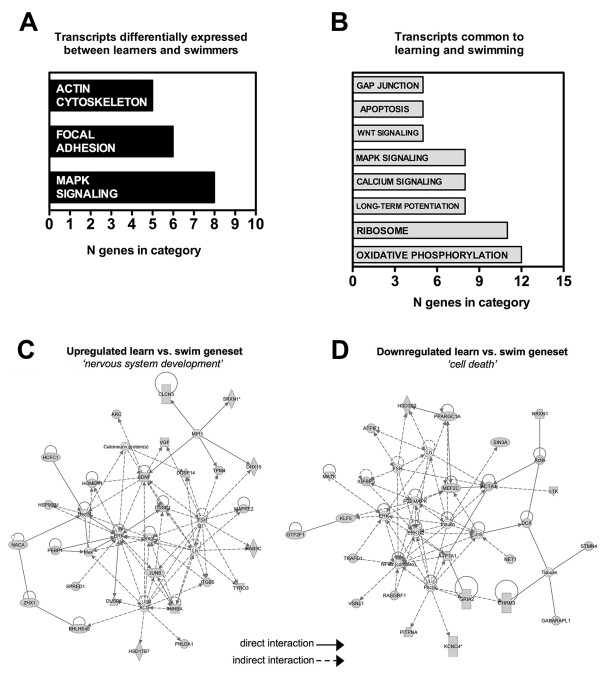
**Gene transcription profiling reveals functional categories regulated by learning and swimming in the cortex of aged mice**. (**A**) Categories of transcripts that were differentially regulated by learning and swimming in 20-month old male C57Bl/6 mice. Signaling pathways implicated in learning, such as the mitogen-activated protein kinase signaling cascade, are prominent among these transcripts. (**B**) Functional pathways activated following learning and swimming. These pathways were derived from analysis using the Kyoto Encyclopedia of Genes and Genomes (KEGG) database. (**A**), Learners upregulated transcripts that are categorized in an unbiased manner by Ingenuity Pathway Analysis as being linked primarily to nervous system development. (**B**), The network derived from genes downregulated in learners, relative to swimmers, is linked primarily to cell death.

We employed signaling pathway enrichment analysis using the pre-determined functional signaling systems delineated by the Kyoto Encyclopedia of Genes and Genomes (KEGG; Figure 3A-B; Table 1). Signaling pathway analysis of the geneset differentially regulated in the learners compared to the swimmers revealed the significant population of pathways related to synaptic architecture (*Actin cytoskeleton*, *Focal adhesion*), energy regulation (*Insulin signaling*), neuronal developmental (*Wnt signaling*) as well as both crucial components of learning and memory (*MAPK signaling*). These pathways have repeatedly been linked with the modulation of learning and memory in the hippocampus [7,22,23]. Therefore mice that exhibit behavioral proficiency in the spatial learning task concurrently upregulated cortical neurotrophic factor expression, and increased activation of cellular signaling pathways associated with long-term potentiation.

**Table 1:** Significantly populated KEGG signaling pathways created by genes differentially regulated between learners and swimmers

KEGG Category	Transcripts	Fold change	p-value
MAPK signaling	brain derived neurotrophic factor	1.4041	0.0063
	growth arrest and DNA-damage-inducible 45 alpha	1.2334	0.0000
	dual specificity phosphatase 1	1.6577	0.0005
	RAS related protein 1b	1.2067	0.0306
	activin A receptor, type IC	1.3618	0.0000
	dual specificity phosphatase 14	1.1473	0.0035
	serine/threonine kinase 4	1.1598	0.0316
	dual specificity phosphatase 6	1.5402	0.0000
Focal adhesion	catenin (cadherin associated protein), beta 1	1.2247	0.0051
	cyclin D1	1.4578	0.0019
	integrin beta 5	1.1444	0.0365
	3-phosphoinositide dependent protein kinase-1	1.1971	0.0211
	protein phosphatase 1, catalytic subunit, beta isoform	1.3220	0.0193
	RAS related protein 1b	1.2067	0.0306
Actin cytoskeleton	integrin beta 5	1.1444	0.0365
	myosin, heavy polypeptide 9, non-muscle	1.3739	0.0061
	protein phosphatase 1, catalytic subunit, beta isoform	1.3220	0.0193
	NCK-associated protein 1	1.1491	0.0427
	actin related protein 2/3 complex, subunit 5		
Insulin signaling	3-phosphoinositide dependent protein kinase-1	1.1971	0.0211
	protein phosphatase 1, catalytic subunit, beta isoform	1.3220	0.0193
	insulin receptor substrate 2	1.3744	0.0229
	protein phosphatase 1, regulatory (inhibitor) subunit 3C	1.2373	0.0329
Wnt signaling	catenin (cadherin associated protein), beta 1	1.2247	0.0051
	cyclin D1	1.4578	0.0019
	protein phosphatase 2, regulatory subunit B, gamma isoform	1.2332	0.0396
	protein phosphatase 2, regulatory subunit B, beta isoform	1.2616	0.0191

The table indicates the most significantly populated KEGG pathways and the corresponding genes from the input dataset (significantly regulated in learners versus swimmers) that were clustered into the respective pathways.

Because the differential learning versus swimming geneset presented a unique insight into the molecular mechanisms of learning and memory in the aged cortex, we decided to further investigate the nature of the actual gene interactions in the cortex that may underpin the coherent regulation of memory formation. We identified the most likely gene interaction networks in the up- and down-regulated genesets using Ingenuity Pathway Analysis. The highest scoring network in the upregulated genes was shown to be linked primarily to '*nervous system development*' (IPA-Network Analysis terminology) (Figure 3C), while the highest scoring network from the downregulated component was linked primarily to '*cell death*' (IPA-Network Analysis terminology) (Figure 3D). This downregulated 'cell death' targeting network demonstrated multiple interactions with genes linked to neuronal pathology including; p38 mitogen-activated protein kinase, Jun N-terminal kinase (JNK) and NF-κB [24]. In contrast, the 'nervous system development' network demonstrated multiple local interactions between known neurotrophic, protective and plasticity-related factors including BDNF, Homer, Vegf, Dusp1, JunB, Vgf and Arc [6,7,24-28]. In this regard, our analysis of learning-induced changes in gene transcription revealed decreases in cell-death associated pathways, and reinstatement or maintenance of a neurodevelopmental signaling program in the aged mouse cortex.

### Cortical distribution of Arc mRNA following learning and swimming

**Figure 4:**
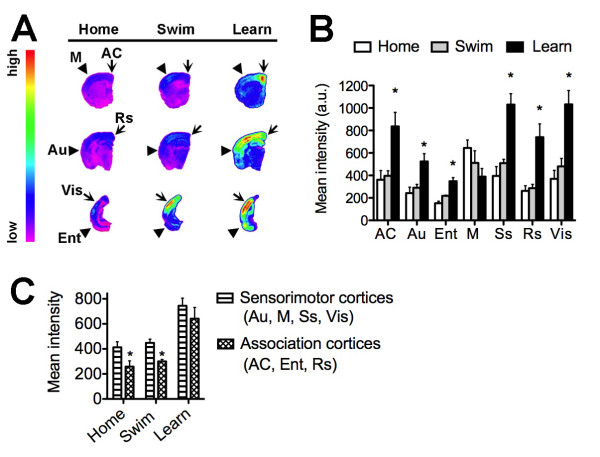
**Patterns of cortical Arc expression following learning in aged mice**. (**A**), Representative heat maps showing the distribution of Arc mRNA expression in the aged mouse brain following learning, swimming, or home cage conditions. (**B**), Densitometric analysis revealed that learning increased Arc expression in the anterior cingulate cortex (AC), auditory cortex (Au), entorhinal cortex (Ent), somatosensory cortex (Ss), retrosplenial cortex (Rs), and visual cortex (Vis). The only cortical region that did not show increased Arc expression following learning was the motor cortex (M). Asterisk (*) indicates significance at p < 0.05 following one-way ANOVA with Tukey's post hoc. Abbreviations used in (**A**) are the same as those used in (**B**) and (**C**); see Materials and Methods for detailed description of cortical anatomical sampling. (**C**), Levels of Arc mRNA are lower in association cortices, relative to sensorimotor regions, under home cage conditions and following swimming without a target in the maze. Following learning, this difference is no longer significant, suggestive of changes in the pattern of cortical network activity.

The gene encoding Arc emerged as a significant hallmark of learning in the microarray data and gene network analysis, and we further characterized the regulation of Arc expression in the aged mouse cortex using in situ hybridization (Figure 4A-B). The anterior cingulate cortex exhibits increased Arc expression following learning (F_2,16 _= 8.051, p = 0.0047; post hoc comparison, learn vs. home cage, t_10 _= 3.16, p = 0.01). In parallel with this observation, the entorhinal cortex also responded to learning with an increase in Arc mRNA (F_2,16 _= 19.75, p = 0.0001, post hoc comparison, learn vs. home cage, t_10 _= 5.26, p = 0.0004). Also within the limbic circuitry, learning enhanced Arc expression in the retrosplenial cortex (F_2,16 _= 11.80, p = 0.001, post hoc comparison, learn vs. home cage, t_10 _= 3.77, p = 0.0036). Among the sensory cortices, learning intensified the Arc signal in the somatosensory cortex (F_2,16 _= 18.77, p = 0.0001, post hoc comparison, learn vs. home cage, t_10 _= 5.007, p = 0.0005), visual cortex (F_2,16 _= 14.46, p = 0.0004, post hoc comparison, learn vs. home cage, t_10 _= 4.61, p = 0.001), and auditory cortex (F_2,16 _= 7.64, p = 0.006, post hoc comparison, learn vs. home cage, t_10 _= 3.24, p = 0.008). Neither learning nor swimming induced Arc expression in the motor cortex (F_2,16 _= 2.55, p = 0.11). Outside of the motor cortex, Arc expression is selectively potentiated by the goal-directed learning task compared to the non-goal-directed swimming task.

To determine whether regions associated with memory networks might respond differently than regions associated with sensorimotor function, we compared the expression of Arc in cortical regions with associational or sensorimotor characteristics. Home cage controls and swimmers both had lower levels of Arc in associational regions (anterior cingulate, entorhinal, and retrosplenial cortices) relative to sensorimotor regions (auditory, motor, somatosensory, and visual cortices). In contrast, levels of Arc expression in sensorimotor and association cortices were similar following learning (Figure 4C). This suggests that learning proficiency alters patterns of network activity in the aged mouse cortex by changing the ratio of activation in sensorimotor and associational regions.

### Homogeneous upregulation of cortical BDNF following learning in aged mice

**Figure 5:**
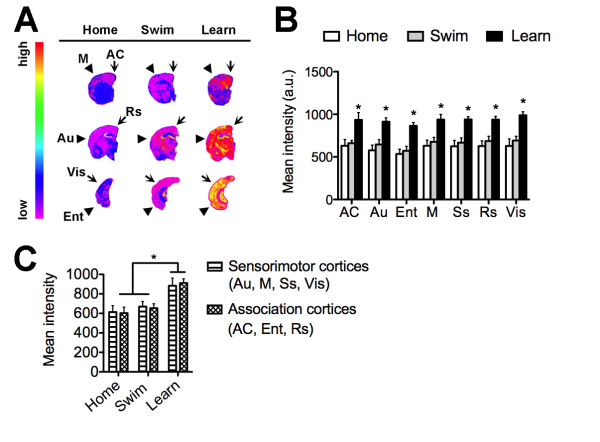
**Homogeneous upregulation of cortical BDNF expression following learning in aged mice**. (**A**), Representative heat maps showing the pattern of BDNF mRNA expression in the aged mouse brain following learning, swimming, or home cage conditions. (**B**), Densitometric analysis revealed that learning enhanced BDNF expression in the anterior cingulate cortex (AC), auditory cortex (Au), entorhinal cortex (Ent), motor cortex (M), somatosensory cortex (Ss), retrosplenial cortex (Rs), and visual cortex (Vis). Asterisk (*) indicates significance at p < 0.05 following one-way ANOVA with Tukey's post hoc. Abbreviations used in (**A**) are the same as those used in (**B**) and (**C**); see Materials and Methods for detailed description of cortical anatomical sampling. (**C**), Levels of BDNF mRNA are comparable across regions involved in the associational and sensorimotor aspects of water maze training.

Cortical BDNF expression increased significantly in our microarray analysis. We used in situ hybridization to validate this observation and further evaluate changes in cortical BDNF levels with greater anatomical specificity. Learning elevated BDNF mRNA expression across all regions examined (Figure 5A-B), including the anterior cingulate (F_2,16 _= 6.11, p = 0.01, post hoc comparison, learn vs. home cage, t_10 _= 2.71, p = 0.02), auditory cortex (F_2,16 _= 9.37, p = 0.002, post hoc comparison, learn vs. home cage, t_10 _= 4.23, p = 0.002), entorhinal cortex (F_2,13 _= 13.75, p = 0.001, post hoc comparison, learn vs. home cage, t_10 _= 4.82, p = 0.001), motor cortex (F_2,16 _= 7.13, p = 0.007, post hoc comparison, learn vs. home cage, t_10 _= 3.38, p = 0.008), somatosensory cortex (F_2,16 _= 8.47, p = 0.004, post hoc comparison, learn vs. home cage, t_10 _= 3.87, p = 0.003), retrosplenial cortex (F_2,16 _= 8.48, p = 0.004, post hoc comparison, learn vs. home cage, t_10 _= 4.08, p = 0.002), and visual cortex (F_2,15 _= 9.92, p = 0.002, post hoc comparison, learn vs. home cage, t_10 _= 3.86, p = 0.004). The relationship between BDNF mRNA expression in sensorimotor and association cortices was unchanged following learning, due to similar upregulation across cortical modules related to the different components of water maze training (Figure 5C). Homogeneous upregulation of cortical BDNF expression following learning aligns with the diverse contributions of BDNF to plasticity across multiple cortical circuits [27,28]. Because BDNF was uniformly upregulated, while Arc expression was more heterogeneous, there may be region-specific mechanisms dissociating BDNF expression from Arc induction in mnemonic versus non-mnemonic circuits.

### Aged mice exhibit reduced cortical GRIT mRNA expression following water maze training

**Figure 6:**
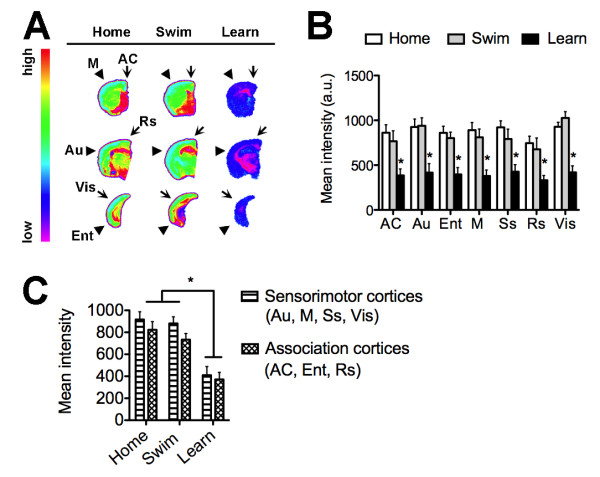
**Widespread downregulation of cortical GRIT expression following learning in aged mice**. (**A**), Representative heat maps showing the distribution of GRIT mRNA expression in the aged mouse brain following learning, swimming, or home cage conditions. (**B**), Densitometric analysis revealed that learning suppressed GRIT expression in all cortical regions examined, including the anterior cingulate cortex (AC), auditory cortex (Au), entorhinal cortex (Ent), motor cortex (M), somatosensory cortex (Ss), retrosplenial cortex (Rs), and visual cortex (Vis). Asterisk (*) indicates significance at p < 0.05 following one-way ANOVA with Tukey's post hoc. Abbreviations used in (**A**) are the same as those used in (**B**) and (**C**); see Materials and Methods for detailed description of cortical anatomical sampling. (**C**), Levels of GRIT mRNA are reduced following learning, to a similar extent in cortical regions implicated in the associational and sensorimotor aspects of water maze learning.

The gene encoding Rho GTPase activating protein 32 (GRIT) has been implicated in synaptic plasticity via regulation of the actin cytoskeleton [29]. Multiple lines of evidence have indicated a close functional relationship between Rho catalytic activity (modulated by multiple Rho GTPase activating proteins) and dynamic cytoskeletal remodelling events in the central nervous system [30-32]. GRIT expression was significantly downregulated following learning in the microarray analysis. We used in situ hybridization to further characterize changes in GRIT expression across multiple cortical regions. Consistent with the microarray data, levels of mRNA encoding GRIT were reduced in learners across the anterior cingulate cortex (F_2,14 _= 7.27, p = 0.008, post hoc comparison, learn vs. home cage, t_10 _= 4.15, p = 0.003), auditory cortex (F_2,12 _= 10.08, p = 0.004, post hoc comparison, learn vs. home cage, t_10 _= 3.82, p = 0.005), entorhinal cortex (F_2,12 _= 12.02, p = 0.002, post hoc comparison, learn vs. home cage, t_10 _= 4.36, p = 0.002), motor cortex (F_2,14 _= 11.22, p = 0.002, post hoc comparison, learn vs. home cage, t_10 _= 4.74, p = 0.001), somatosensory cortex (F_2,14 _= 8.36, p = 0.005, post hoc comparison, learn vs. home cage, t_10 _= 4.61, p = 0.001), retrosplenial cortex (F_2,14 _= 5.99, p = 0.016, post hoc comparison, learn vs. home cage, t_10 _= 4.43, p = 0.002), and visual cortex (F_2,14 _= 25.38, p = 0.001, post hoc comparison, learn vs. home cage, t_10 _= 5.77, p = 0.0004) (Figure 6A-B). Downregulation of GRIT occurred to a similar extent in sensorimotor and association cortices (Figure 6C), suggesting that suppression of this transcript is involved in multiple aspects of water maze learning.

## Discussion

We have performed transcriptional analysis in order to investigate the regulation of cortical gene expression by learning in the aged mouse. The cortical transcriptional profiles and their associated signaling pathways suggest that many molecular and cellular properties related to the enhancement of synaptic plasticity were induced following learning. Features specific to learning induce Arc and BDNF expression, and suppress GRIT transcription across multiple regions of the aged cortex. The widespread transcriptional response to learning supports a role for mental activity in the regulation of genes that support synaptic function in the aging brain.

The transcriptional response to learning was comprised mainly of genes that met our statistical criteria for upregulation. This contrasts with the previous observation that hippocampal gene expression patterns following learning and swimming were comparably attributable to bi-directional transcriptional regulation [7]. This difference might reflect the distinct contributions of transcriptional mechanisms in the hippocampus and cortex to associative memory formation. While the hippocampus contributes to the acquisition of spatial memories in the water maze, the associative representation of the platform location is presumably stored in the cortex. As tissue samples were taken after six days of training, when animals had reached asymptotic performance, it is possible that the trend for transcriptional upregulation that we observed in our microarray data is attributable to distributed memory storage across multiple cortical regions.

Our microarray data is a summation of distinct transcriptional mechanisms occurring in different areas of the cortex. We plan eventually to conduct additional further studies to build on our current findings by investigating distinct mechanisms of transcriptional activity in multiple smaller-scale cortical regions, including separate analysis of primary and secondary sensory cortices. However, it is apparent from our in situ hybridization analysis that most cortical regions respond to six days of training with an increase in Arc and BDNF expression, and a decrease in GRIT mRNA. To the extent that Arc, BDNF, and GRIT can be taken as general indicators of synaptic plasticity, it then becomes possible to speculate that multiple regions of cortex respond to learning with changes in synaptic function, as observed in our network analyses of global gene expression. In this regard, our in situ hybridization results, which possess strong anatomical resolution, are consistent with our microarray results, which used samples derived from global cortical RNA. While our transcriptional analysis has generated a broad appreciation of genomic responses to learning activities, such analyses alone may not give the clearest view of the neurophysiological responses to learning activities. An even more complete picture of such neurophysiological responses may be gained by performing additional investigations into the quantitative proteomic responses to similar behavioral paradigms. Therefore the complementary analyses of both the quantitative genomic and proteomic responses to these behavioral paradigms may, in the future, give the best overview of the myriad complex and subtle neuronal processes involved in spatial learning.

Using gene network analysis, we were further able to identify key factors for the learning-specific reactive mechanisms in the aged cortex. Arc induction was specific to learning, as shown by both microarray and in situ hybridization. This suggests that certain genes (such as Arc) distinguish between goal-directed learning and non-goal-directed swimming. This more detailed inspection of the transcriptional distinctions of learning-induced cortical changes demonstrated a coherent response to the challenge of a targeted task by promoting increasing association of pro-neurotrophic agents, while simultaneously attenuating more damaging functional mechanisms. Hence, the most likely functional gene network created using the learning downregulated geneset was involved in regulating cell death mechanisms. The gene network most likely to statistically exist among the genes upregulated in a learning-specific manner possessed a strong developmental phenotype. This network comprised of multiple neurotrophic and plasticity-related factors, such as BDNF, Vgf and Arc. Arc synthesis is necessary for consolidation of hippocampal LTP and the formation of spatial memories in the water maze [33]. Widespread induction of cortical Arc in learners relative to swimmers is consistent with previous data from young rats [34], and implies that LTP is potentially induced across multiple regions of the aged mouse cortex by water maze training. Also, based on differences in the expression of BDNF and Vgf between learners and swimmers, growth factor expression seems to be induced by cognitive stimulation rather than swimming without a target in the maze.

## Conclusions

Cognitive stimulation is critical for healthy aging. As shown in the current study, aged mice that retain the capacity for effective learning also increase their expression of plasticity-related genes following cognitive stimulation. While the functional contributions of changes in cortical gene transcription to learning across the lifespan remain to be determined, understanding the transcriptional response to learning could potentially pave the way for novel therapies that can maintain or enhance cognitive function during aging.

## Authors' contributions

SSP, AMS and SM designed research; SSP, AMS, WC, YZ, LW, BM and KGB performed research, and all authors participated in writing the paper. All authors read and approved the final manuscript.

## Supplementary Material

Additional file 1 **Gene transcripts differentially and significantly regulated between learning and swimming task mice**. The table indicates the gene transcripts significantly and differentially regulated in the cortex of mice performing a learning task, involving Morris Water Maze completion, compared to mice performing a controlled swimming task alone. Click here for file
