# Supplementary material for: Cortical gene transcription response patterns to water maze training in aged mice
Source: BMC Neurosci. 2011 Jun 29;12:63. doi: 10.1186/1471-2202-12-63 (PMC3142531; doi:10.1186/1471-2202-12-63)
Supplement: Additional file 1 — Gene transcripts differentially and significantly regulated between learning and swimming task mice. The table indicates the gene transcripts significantly and differentially regulated in the cortex of mice performing a learning task, involving Morris Water Maze completion, compared to mice performing a controlled swimming task alone. [file 1471-2202-12-63-S1.DOC]

**Table S1.** Genes that differentiate learning and swimming in the cortex of aged mice. Genes differentially regulated in learning process animals compared to swimming process animals are indicated. For each significantly regulated transcript the gene description is followed by the official gene symbol in parentheses. For each transcript the normalized z ratio, probability value and fold change (learners relative to swimmers) are indicated.

| **Gene Description (Gene Symbol)** | **z ratio** | **p value** | **fold change** |
| --- | --- | --- | --- |
| activity regulated cytoskeletal-associated protein (Arc) | 8.04 | 0.00 | 2.24 |
| dual specificity phosphatase 1 (Dusp1) | 4.69 | 0.00 | 1.66 |
| inhibin beta-A (Inhba) | 4.66 | 0.00 | 1.58 |
| troponin C, cardiac/slow skeletal (Tnnc1) | 4.59 | 0.00 | 1.61 |
| early growth response 4 (Egr4) | 4.11 | 0.01 | 1.53 |
| tescalcin (Tesc) | 3.99 | 0.00 | 1.49 |
| glucosaminyl (N-acetyl) transferase 2, I-branching enzyme (Gcnt2), transcript variant 3 | 3.86 | 0.00 | 1.54 |
| IQ motif containing GTPase activating protein 2 (Iqgap2) | 3.86 | 0.01 | 1.45 |
| family with sequence similarity 40, member B (Fam40b) | 3.83 | 0.02 | 1.49 |
| dual specificity phosphatase 6 (Dusp6) | 3.71 | 0.00 | 1.54 |
| zinc finger, DHHC domain containing 14 (Zdhhc14) | 3.52 | 0.00 | 1.46 |
| cyclin D1 (Ccnd1) | 3.41 | 0.00 | 1.46 |
| Jun-B oncogene (Junb) | 3.38 | 0.00 | 1.40 |
| brain derived neurotrophic factor (Bdnf), transcript variant 3 | 3.37 | 0.01 | 1.40 |
| activin A receptor, type IC (Acvr1c) | 3.34 | 0.00 | 1.36 |
| diacylglycerol kinase, beta (Dgkb) | 3.25 | 0.00 | 1.41 |
| RIKEN cDNA 6330578E17 gene (6330578E17Rik) | 3.19 | 0.00 | 1.43 |
| protein phosphatase 1, catalytic subunit, beta isoform (Ppp1cb) | 3.13 | 0.01 | 1.35 |
| F-box protein 3 (Fbxo3), transcript variant 1 | 3.10 | 0.00 | 1.38 |
| ubiquitin carboxyl-terminal esterase L3 (ubiquitin thiolesterase) (Uchl3) | 3.04 | 0.01 | 1.33 |
| ubiquitin carboxyl-terminal esterase L5 (Uchl5) | 3.03 | 0.04 | 1.31 |
| ecto-NOX disulfide-thiol exchanger 1 (Enox1) | 2.94 | 0.00 | 1.34 |
| schwannomin interacting protein 1 (Schip1) | 2.93 | 0.00 | 1.38 |
| RIKEN cDNA 4833439L19 gene (4833439L19Rik) | 2.90 | 0.02 | 1.33 |
| nudix (nucleoside diphosphate linked moiety X)-type motif 5 (Nudt5) | 2.89 | 0.00 | 1.30 |
| cytochrome b5 reductase 4 (Cyb5r4) | 2.85 | 0.02 | 1.32 |
| cell cycle associated protein 1 (Caprin1) | 2.85 | 0.00 | 1.30 |
| V-set and transmembrane domain containing 2B (Vstm2b) | 2.83 | 0.00 | 1.39 |
| ADP-ribosylation factor-like 3 (Arl3) | 2.83 | 0.00 | 1.36 |
| cell cycle associated protein 1 (Caprin1) | 2.82 | 0.01 | 1.32 |
| OTU domain, ubiquitin aldehyde binding 2 (Otub2) | 2.81 | 0.00 | 1.29 |
| insulin receptor substrate 2 (Irs2) | 2.81 | 0.02 | 1.37 |
| protein phosphatase 2C, magnesium dependent, catalytic subunit (Ppm2c), nuclear gene encoding mitochondrial protein, transcript variant 1 | 2.79 | 0.02 | 1.32 |
| myosin, light polypeptide 4 (Myl4) | 2.76 | 0.01 | 1.37 |
| centaurin, delta 3 (Centd3) | 2.76 | 0.00 | 1.29 |
| tropomyosin 4 (Tpm4) | 2.73 | 0.00 | 1.31 |
| vesicle transport through interaction with t-SNAREs 1B homolog (Vti1b) | 2.72 | 0.02 | 1.31 |
| synaptotagmin XIII (Syt13) | 2.65 | 0.00 | 1.26 |
| influenza virus NS1A binding protein (Ivns1abp), transcript variant 2 | 2.64 | 0.00 | 1.34 |
| RIKEN cDNA 6530418L21 gene (6530418L21Rik) | 2.61 | 0.00 | 1.27 |
| histone cluster 2, H3b (Hist2h3b) | 2.58 | 0.00 | 1.27 |
| potassium channel tetramerisation domain containing 12 (Kctd12) | 2.55 | 0.00 | 1.25 |
| ubiquitin-conjugating enzyme E2F (putative) (Ube2f) | 2.54 | 0.00 | 1.27 |
| factor 8-associated gene A (F8a) | 2.54 | 0.00 | 1.30 |
| ubiquitin-conjugating enzyme E2G 1 (UBC7 homolog, C. elegans) (Ube2g1) | 2.54 | 0.01 | 1.27 |
| abl-interactor 1 (Abi1), transcript variant 1 | 2.53 | 0.00 | 1.32 |
| basic helix-loop-helix domain containing, class B2 (Bhlhb2) | 2.52 | 0.01 | 1.31 |
| VGF nerve growth factor inducible (Vgf) | 2.50 | 0.00 | 1.36 |
| zinc finger protein 238 (Zfp238), transcript variant 2 | 2.49 | 0.00 | 1.29 |
| protein phosphatase 2 (formerly 2A), regulatory subunit B (PR 52), gamma isoform (Ppp2r2c) | 2.42 | 0.02 | 1.26 |
| SEC63-like (Sec63) | 2.42 | 0.00 | 1.26 |
| proteasome (prosome, macropain) 26S subunit, non-ATPase, 7 (Psmd7) | 2.41 | 0.03 | 1.28 |
| mitochondrial ribosomal protein S18C (Mrps18c), nuclear gene encoding mitochondrial protein | 2.41 | 0.00 | 1.26 |
| cornichon homolog (Cnih) | 2.40 | 0.01 | 1.25 |
| small nuclear RNA activating complex, polypeptide 3 (Snapc3) | 2.40 | 0.00 | 1.24 |
| transcription factor A, mitochondrial (Tfam) | 2.40 | 0.00 | 1.23 |
| trophoblast glycoprotein (Tpbg) | 2.38 | 0.00 | 1.27 |
| methyltransferase like 9 (Mettl9) | 2.37 | 0.01 | 1.26 |
| RAP2C, member of RAS oncogene family (Rap2c) | 2.37 | 0.04 | 1.25 |
| trafficking protein particle complex 2 (Trappc2) | 2.37 | 0.00 | 1.24 |
| transmembrane protein 128 (Tmem128) | 2.36 | 0.02 | 1.28 |
| tribbles homolog 2 (Trib2) | 2.36 | 0.00 | 1.25 |
| growth arrest and DNA-damage-inducible 45 alpha (Gadd45a) | 2.35 | 0.00 | 1.23 |
| RAP2C, member of RAS oncogene family (Rap2c) | 2.34 | 0.01 | 1.26 |
| mediator complex subunit 7 (Med7) | 2.32 | 0.00 | 1.26 |
| acyl-CoA thioesterase 1 (Acot1) | 2.31 | 0.01 | 1.27 |
| oligosaccharyltransferase complex subunit (Ostc) | 2.29 | 0.00 | 1.31 |
| chloride channel 3 (Clcn3), transcript variant a | 2.28 | 0.02 | 1.25 |
| transmembrane protein 66 (Tmem66) | 2.27 | 0.05 | 1.25 |
| thyroid hormone receptor interactor 12 (Trip12) | 2.26 | 0.00 | 1.23 |
| glypican 1 (Gpc1) | 2.25 | 0.02 | 1.36 |
| doublecortin-like kinase 3 (Dclk3) | 2.24 | 0.01 | 1.27 |
| heat shock protein 1 (chaperonin) (Hspd1) | 2.23 | 0.00 | 1.29 |
| SH3-domain kinase binding protein 1 (Sh3kbp1) | 2.23 | 0.04 | 1.23 |
| resistance to inhibitors of cholinesterase 8 homolog B (Ric8b), transcript variant 2 | 2.23 | 0.04 | 1.24 |
| eukaryotic translation elongation factor 1 epsilon 1 (Eef1e1) | 2.22 | 0.00 | 1.23 |
| polymerase (RNA) II (DNA directed) polypeptide G (Polr2g) | 2.21 | 0.00 | 1.25 |
| plastin 3 (T-isoform) (Pls3) | 2.20 | 0.00 | 1.22 |
| kelch-like 9 (Klhl9) | 2.20 | 0.02 | 1.21 |
| Ras and Rab interactor 1 (Rin1) | 2.19 | 0.00 | 1.25 |
| ankyrin repeat domain 46 (Ankrd46) | 2.19 | 0.02 | 1.24 |
| RNA U, small nuclear RNA export adaptor (Rnuxa) | 2.19 | 0.00 | 1.21 |
| DnaJ (Hsp40) homolog, subfamily C, member 15 (Dnajc15) | 2.18 | 0.03 | 1.28 |
| golgi membrane protein 1 (Golm1), transcript variant 1 | 2.18 | 0.00 | 1.23 |
| neuronal pentraxin 2 (Nptx2) | 2.18 | 0.00 | 1.28 |
| insulin-like growth factor binding protein 7 (Igfbp7) | 2.18 | 0.02 | 1.25 |
| transmembrane protein 33 (Tmem33), transcript variant 2 | 2.17 | 0.00 | 1.26 |
| ornithine decarboxylase, structural 1 (Odc1) | 2.15 | 0.00 | 1.27 |
| histone cluster 2, H3c1 (Hist2h3c1), transcript variant 1 | 2.14 | 0.00 | 1.21 |
| growth hormone inducible transmembrane protein (Ghitm) | 2.12 | 0.05 | 1.23 |
| zinc finger matrin type 3 (Zmat3) | 2.12 | 0.02 | 1.26 |
| tetraspanin 14 (Tspan14) | 2.12 | 0.00 | 1.24 |
| SH3-binding kinase 1 (Sbk) | 2.09 | 0.00 | 1.28 |
| SMT3 suppressor of mif two 3 homolog 1 (Sumo1) | 2.09 | 0.00 | 1.23 |
| midnolin (Midn) | 2.07 | 0.03 | 1.28 |
| cysteine-rich hydrophobic domain 2 (Chic2) | 2.07 | 0.00 | 1.20 |
| ATP-binding cassette, sub-family F (GCN20), member 2 (Abcf2), nuclear gene encoding mitochondrial protein | 2.06 | 0.01 | 1.22 |
| proteasome (prosome, macropain) 26S subunit, non-ATPase, 10 (Psmd10) | 2.06 | 0.00 | 1.23 |
| heat shock protein 90, beta (Grp94), member 1 (Hsp90b1) | 2.06 | 0.01 | 1.28 |
| phenylalkylamine Ca2+ antagonist (emopamil) binding protein (Ebp) | 2.05 | 0.00 | 1.19 |
| TYRO3 protein tyrosine kinase 3 (Tyro3) XM_925062 | 2.04 | 0.04 | 1.20 |
| protein phosphatase 2 (formerly 2A), regulatory subunit B (PR 52), beta isoform (Ppp2r2b), transcript variant 2 | 2.04 | 0.04 | 1.23 |
| synaptobrevin like 1 (Sybl1) | 2.04 | 0.04 | 1.20 |
| V-set and transmembrane domain containing 2A (Vstm2a) | 2.03 | 0.00 | 1.23 |
| ATPase, H+ transporting, lysosomal V1 subunit A (Atp6v1a) | 2.03 | 0.01 | 1.25 |
| progestin and adipoQ receptor family member IX (Paqr9) | 2.03 | 0.00 | 1.19 |
| phosphatidylethanolamine binding protein 1 (Pebp1) | 2.02 | 0.00 | 1.20 |
| cysteine dioxygenase 1, cytosolic (Cdo1) | 2.02 | 0.05 | 1.21 |
| calcium/calmodulin-dependent protein kinase II inhibitor 1 (Camk2n1), mRNA. | 2.01 | 0.00 | 4.54 |
| sulfiredoxin 1 homolog (Srxn1) | 2.01 | 0.00 | 1.24 |
| karyopherin (importin) alpha 1 (Kpna1) | 2.01 | 0.00 | 1.22 |
| transmembrane protein 33 (Tmem33), transcript variant 1 | 2.00 | 0.00 | 1.24 |
| poly (A) polymerase alpha (Papola) | 1.99 | 0.01 | 1.20 |
| prion protein (Prnp), mRNA. | 1.99 | 0.00 | 4.39 |
| polymerase (DNA-directed), delta interacting protein 3 (Poldip3) | 1.98 | 0.02 | 1.24 |
| golgi membrane protein 1 (Golm1), transcript variant 2 | 1.98 | 0.03 | 1.19 |
| RAB5C, member RAS oncogene family (Rab5c) | 1.96 | 0.04 | 1.23 |
| pleckstrin homology-like domain, family A, member 1 (Phlda1) | 1.95 | 0.00 | 1.22 |
| cytidine monophospho-N-acetylneuraminic acid synthetase (Cmas) | 1.94 | 0.01 | 1.23 |
| transcriptional adaptor 1 (HFI1 homolog, yeast) like (Tada1l) | 1.93 | 0.01 | 1.20 |
| ADP-ribosylation factor-like 8A (Arl8a) | 1.92 | 0.00 | 1.27 |
| NCK-associated protein 1 (Nckap1) | 1.91 | 0.01 | 1.27 |
| thioredoxin-like 4A (Txnl4a), transcript variant 1 | 1.90 | 0.00 | 1.22 |
| ubiquitin specific peptidase 39 (Usp39) | 1.89 | 0.01 | 1.18 |
| roundabout homolog 1 (Robo1) | 1.88 | 0.00 | 1.18 |
| complement component 1, q subcomponent, C chain (C1qc) | 1.88 | 0.01 | 1.21 |
| chemokine (C-X-C motif) ligand 12 (Cxcl12) | 1.87 | 0.00 | 1.25 |
| histone cluster 1, H2bf (Hist1h2bf) | 1.87 | 0.05 | 1.23 |
| ARP3 actin-related protein 3 homolog (Actr3) | 1.87 | 0.00 | 1.24 |
| glycine receptor, beta subunit (Glrb) | 1.87 | 0.00 | 1.17 |
| tetratricopeptide repeat domain 1 (Ttc1) | 1.86 | 0.00 | 1.21 |
| immediate early response 3 (Ier3) | 1.85 | 0.00 | 1.20 |
| ACN9 homolog (Acn9) | 1.84 | 0.02 | 1.18 |
| hydroxysteroid (17-beta) dehydrogenase 7 (Hsd17b7) | 1.83 | 0.03 | 1.23 |
| homer homolog 1 (Homer1), transcript variant d | 1.83 | 0.02 | 1.26 |
| Ngfi-A binding protein 1 (Nab1) | 1.83 | 0.04 | 1.16 |
| 3-phosphoinositide dependent protein kinase-1 (Pdpk1), transcript variant 2 | 1.82 | 0.02 | 1.20 |
| engulfment and cell motility 2, ced-12 homolog (Elmo2), transcript variant 3 | 1.81 | 0.01 | 1.17 |
| Mki67 (FHA domain) interacting nucleolar phosphoprotein (Mki67ip) | 1.80 | 0.00 | 1.17 |
| mediator of RNA polymerase II transcription, subunit 28 homolog (Med28) | 1.80 | 0.02 | 1.19 |
| zinc ribbon domain containing, 1 (Znrd1) | 1.79 | 0.04 | 1.15 |
| cAMP-regulated phosphoprotein 19 (Arpp19) | 1.78 | 0.01 | 1.23 |
| autophagy-related 5 (Atg5) | 1.78 | 0.00 | 1.18 |
| peroxisome biogenesis factor 2 (Pex2) | 1.77 | 0.04 | 1.18 |
| insulin induced gene 2 (Insig2) | 1.77 | 0.01 | 1.17 |
| smu-1 suppressor of mec-8 and unc-52 homolog (Smu1) | 1.76 | 0.01 | 1.18 |
| inositol 1,3,4,5,6-pentakisphosphate 2-kinase (Ippk) | 1.75 | 0.00 | 1.17 |
| actin related protein 2/3 complex, subunit 5 (Arpc5) | 1.75 | 0.04 | 1.15 |
| fractured callus expressed transcript 1 (Fxc1) | 1.75 | 0.04 | 1.16 |
| zinc finger and BTB domain containing 33 (Zbtb33), transcript variant 2 | 1.75 | 0.00 | 1.17 |
| ectonucleotide pyrophosphatase/phosphodiesterase 2 (Enpp2) | 1.75 | 0.02 | 1.24 |
| cylindromatosis (turban tumor syndrome) (Cyld) | 1.74 | 0.02 | 1.24 |
| split hand/foot malformation (ectrodactyly) type 1 (Shfm1) | 1.74 | 0.01 | 1.23 |
| uridine-cytidine kinase 2 (Uck2) | 1.74 | 0.00 | 1.16 |
| thiosulfate sulfurtransferase, mitochondrial (Tst), nuclear gene encoding mitochondrial protein | 1.74 | 0.03 | 1.17 |
| DEAH (Asp-Glu-Ala-His) box polypeptide 15 (Dhx15), transcript variant 2 | 1.74 | 0.01 | 1.18 |
| mitogen-activated protein kinase kinase kinase 7 interacting protein 3 (Map3k7ip3) | 1.73 | 0.00 | 1.17 |
| suppressor of cytokine signaling 5 (Socs5) | 1.73 | 0.00 | 1.20 |
| nascent polypeptide-associated complex alpha polypeptide (Naca) | 1.73 | 0.00 | 1.19 |
| homocysteine-inducible, endoplasmic reticulum stress-inducible, ubiquitin-like domain member 1 (Herpud1) | 1.73 | 0.00 | 1.21 |
| transcription elongation factor A (SII)-like 8 (Tceal8) | 1.73 | 0.02 | 1.20 |
| endosulfine alpha (Ensa), transcript variant 2 | 1.73 | 0.03 | 1.19 |
| palmdelphin (Palmd) | 1.73 | 0.01 | 1.16 |
| solute carrier family 40 (iron-regulated transporter), member 1 (Slc40a1) | 1.72 | 0.02 | 1.16 |
| zinc fingers and homeoboxes 1 (Zhx1), transcript variant 1 | 1.72 | 0.05 | 1.17 |
| translocase of inner mitochondrial membrane 17a (Timm17a), nuclear gene encoding mitochondrial protein | 1.72 | 0.00 | 1.26 |
| steroid 5 alpha-reductase 3 (Srd5a3) | 1.72 | 0.01 | 1.17 |
| solute carrier family 25 (mitochondrial carrier, brain), member 14 (Slc25a14), nuclear gene encoding mitochondrial protein | 1.72 | 0.00 | 1.18 |
| calmodulin 3 (Calm3) | 1.71 | 0.00 | 3.16 |
| host cell factor C1 (Hcfc1) | 1.70 | 0.00 | 1.17 |
| synaptophysin (Syp), mRNA. | 1.70 | 0.00 | 3.11 |
| Tax1 (human T-cell leukemia virus type I) binding protein 3 (Tax1bp3) | 1.70 | 0.00 | 1.15 |
| protein phosphatase 1, regulatory (inhibitor) subunit 3C (Ppp1r3c) | 1.70 | 0.03 | 1.24 |
| NHL repeat containing 1 (Nhlrc1) | 1.70 | 0.01 | 1.16 |
| FK506 binding protein 9 (Fkbp9) | 1.69 | 0.02 | 1.17 |
| dynein light chain Tctex-type 3 (Dynlt3) | 1.69 | 0.03 | 1.19 |
| sprouty protein with EVH-1 domain 1, related sequence (Spred1) | 1.69 | 0.01 | 1.25 |
| craniofacial development protein 1 (Cfdp1) | 1.69 | 0.03 | 1.17 |
| RAS related protein 1b (Rap1b) | 1.68 | 0.03 | 1.21 |
| signal recognition particle receptor ('docking protein') (Srpr) | 1.66 | 0.00 | 1.15 |
| solute carrier family 25 (mitochondrial carrier, peroxisomal membrane protein), member 17 (Slc25a17), nuclear gene encoding mitochondrial protein | 1.65 | 0.02 | 1.14 |
| monoglyceride lipase (Mgll) | 1.64 | 0.00 | 1.22 |
| glutamate receptor, ionotropic, N-methyl D-aspartate-associated protein 1 (glutamate binding) (Grina), mRNA. | 1.64 | 0.00 | 2.92 |
| coenzyme Q10 homolog B (Coq10b), transcript variant 1 | 1.64 | 0.04 | 1.17 |
| RAB28, member RAS oncogene family (Rab28) | 1.64 | 0.03 | 1.22 |
| integrin beta 5 (Itgb5) | 1.63 | 0.04 | 1.14 |
| BUD31 homolog (Bud31) | 1.63 | 0.00 | 1.19 |
| ARP10 actin-related protein 10 homolog (Actr10) | 1.63 | 0.00 | 1.21 |
| tubulin, beta 5 (Tubb5) | 1.63 | 0.00 | 2.88 |
| SMT3 suppressor of mif two 3 homolog 2 (Sumo2) | 1.63 | 0.04 | 1.23 |
| catenin (cadherin associated protein), beta 1 (Ctnnb1) | 1.62 | 0.01 | 1.22 |
| electron transferring flavoprotein, dehydrogenase (Etfdh) | 1.62 | 0.04 | 1.13 |
| RNA guanylyltransferase and 5'-phosphatase (Rngtt) | 1.62 | 0.00 | 1.15 |
| myosin, heavy polypeptide 9, non-muscle (Myh9) | 1.61 | 0.01 | 1.17 |
| leucine rich repeat and fibronectin type III domain containing 2 (Lrfn2) | 1.61 | 0.02 | 1.15 |
| lon peptidase 2, peroxisomal (Lonp2) | 1.61 | 0.00 | 1.19 |
| syntaxin 6 (Stx6) | 1.61 | 0.03 | 1.14 |
| transmembrane protein 198 (Tmem198) | 1.60 | 0.00 | 1.18 |
| GC-rich promoter binding protein 1 (Gpbp1) | 1.60 | 0.00 | 1.17 |
| DnaJ (Hsp40) homolog, subfamily C, member 3A (Dnajc3a) | 1.60 | 0.00 | 1.15 |
| exocyst complex component 8 (Exoc8) | 1.60 | 0.01 | 1.19 |
| DPH3 homolog (KTI11, S. cerevisiae) (Dph3), transcript variant 1 | 1.59 | 0.01 | 1.16 |
| microfibrillar-associated protein 1B (Mfap1b) | 1.59 | 0.00 | 1.16 |
| myeloid differentiation primary response gene 88 (Myd88) | 1.58 | 0.00 | 1.14 |
| glutamyl-prolyl-tRNA synthetase (Eprs) | 1.58 | 0.04 | 1.13 |
| tumor necrosis factor receptor superfamily, member 19 (Tnfrsf19) | 1.58 | 0.03 | 1.14 |
| neuroplastin (Nptn) | 1.57 | 0.00 | 2.70 |
| transmembrane protein 77 (Tmem77), transcript variant 2 | 1.57 | 0.00 | 1.14 |
| kinesin-associated protein 3 (Kifap3) | 1.56 | 0.03 | 1.17 |
| solute carrier family 25 (mitochondrial carnitine/acylcarnitine translocase), member 20 (Slc25a20) | 1.56 | 0.00 | 1.15 |
| histone cluster 1, H2bj (Hist1h2bj) | 1.55 | 0.03 | 1.16 |
| serine/threonine kinase 4 (Stk4) | 1.55 | 0.03 | 1.16 |
| dual specificity phosphatase 14 (Dusp14) | 1.55 | 0.00 | 1.15 |
| microfibrillar-associated protein 3 (Mfap3), transcript variant 2 | 1.54 | 0.04 | 1.15 |
| transmembrane protein 185B (Tmem185b) | 1.54 | 0.00 | 1.16 |
| microtubule-associated protein, RP/EB family, member 2 (Mapre2) | 1.54 | 0.00 | 1.18 |
| TSC22 domain family 2 (Tsc22d2) | 1.54 | 0.02 | 1.14 |
| RUN domain containing 3B (Rundc3b) | 1.53 | 0.01 | 1.13 |
| syndecan 2 (Sdc2) | 1.53 | 0.04 | 1.16 |
| nuclear import 7 homolog (Nip7) | 1.53 | 0.00 | 1.14 |
| Unc-51 like kinase 2 (Ulk2) | 1.53 | 0.02 | 1.16 |
| protein disulfide isomerase associated 3 (Pdia3) | 1.53 | 0.00 | 1.22 |
| coenzyme Q2 homolog, prenyltransferase (Coq2) | 1.52 | 0.01 | 1.18 |
| methionine sulfoxide reductase B2 (Msrb2) | 1.52 | 0.01 | 1.17 |
| solute carrier family 11 (proton-coupled divalent metal ion transporters), member 1 (Slc11a1) | 1.52 | 0.02 | 1.12 |
| translocase of inner mitochondrial membrane 10 homolog (Timm10) | 1.52 | 0.00 | 1.13 |
| DEAD (Asp-Glu-Ala-Asp) box polypeptide 20 (Ddx20) | 1.52 | 0.01 | 1.12 |
| synapsin I (Syn1) | 1.51 | 0.00 | 2.51 |
| RIKEN cDNA 6330406I15 gene (6330406I15Rik) | 1.51 | 0.03 | 4.19 |
| F-box protein 33 (Fbxo33) | 1.51 | 0.03 | 1.15 |
| histone deacetylase 5 (Hdac5), transcript variant 1 | 1.51 | 0.04 | 1.19 |
| solute carrier family 35, member F3 (Slc35f3) | 1.50 | 0.02 | 1.17 |
| growth arrest specific 7 (Gas7) | 1.50 | 0.00 | 1.14 |
| gene model 347, (NCBI) (Gm347) | -1.51 | 0.01 | -1.19 |
| beta-1,4-N-acetyl-galactosaminyl transferase 4 (B4galnt4) | -1.51 | 0.00 | -1.23 |
| ATPase, H+ transporting, lysosomal accessory protein 1 (Atp6ap1) | -1.52 | 0.04 | -1.07 |
| bromodomain containing 9 (Brd9) | -1.52 | 0.02 | -1.14 |
| actin, alpha 1, skeletal muscle (Acta1) | -1.52 | 0.00 | -1.22 |
| translocase of inner mitochondrial membrane 44 (Timm44) | -1.52 | 0.04 | -1.22 |
| carbohydrate (N-acetylgalactosamine 4-0) sulfotransferase 8 (Chst8) | -1.53 | 0.03 | -1.18 |
| glutamate receptor, ionotropic, AMPA2 (alpha 2) (Gria2) | -1.54 | 0.04 | -1.08 |
| tubulin, gamma complex associated protein 2 (Tubgcp2) | -1.54 | 0.00 | -1.18 |
| yippee-like 3 (Ypel3) | -1.54 | 0.03 | -1.07 |
| phosphatidylinositol transfer protein, alpha (Pitpna) | -1.55 | 0.00 | -1.12 |
| solute carrier family 4 (anion exchanger), member 3 (Slc4a3) | -1.55 | 0.01 | -1.18 |
| zinc finger, CCHC domain containing 6 (Zcchc6) | -1.55 | 0.00 | -1.17 |
| stathmin-like 4 (Stmn4) | -1.55 | 0.03 | -1.16 |
| testis expressed gene 264 (Tex264), transcript variant 1 | -1.56 | 0.02 | -1.18 |
| Rab6 interacting protein 1 (Rab6ip1) | -1.56 | 0.02 | -1.14 |
| transcriptional regulator, SIN3A (Sin3a) | -1.57 | 0.00 | -1.21 |
| aconitase 2, mitochondrial (Aco2), nuclear gene encoding mitochondrial protein | -1.58 | 0.02 | -1.12 |
| KRAB-A domain containing 1 (Krba1) | -1.59 | 0.00 | -1.22 |
| DEAD (Asp-Glu-Ala-Asp) box polypeptide 3, Y-linked (Ddx3y) | -1.59 | 0.00 | -1.15 |
| myb-like, SWIRM and MPN domains 1 (Mysm1) | -1.60 | 0.03 | -1.19 |
| ribosomal protein S19 (Rps19) | -1.60 | 0.00 | -1.09 |
| transcription elongation factor A (SII), 2 (Tcea2) | -1.61 | 0.02 | -1.16 |
| RIKEN cDNA 1810007P19 gene (1810007P19Rik) | -1.63 | 0.00 | -1.24 |
| RAB6B, member RAS oncogene family (Rab6b) | -1.64 | 0.01 | -1.08 |
| calcium channel, voltage-dependent, alpha2/delta subunit 3 (Cacna2d3) | -1.64 | 0.03 | -1.18 |
| kinesin family member 5C (Kif5c) | -1.67 | 0.04 | -1.10 |
| general transcription factor IIF, polypeptide 1 (Gtf2f1) | -1.67 | 0.05 | -1.25 |
| enolase 2, gamma neuronal (Eno2) | -1.67 | 0.03 | -1.07 |
| ribosomal protein S21 (Rps21) | -1.67 | 0.03 | -1.07 |
| insulin-like growth factor binding protein 5 (Igfbp5) | -1.68 | 0.00 | -1.15 |
| neuroepithelial cell transforming gene 1 (Net1), transcript variant 1 | -1.68 | 0.01 | -1.26 |
| axin2 (Axin2) | -1.68 | 0.01 | -1.23 |
| cyclic AMP-regulated phosphoprotein, 21 (Arpp21), transcript variant 2 | -1.68 | 0.03 | -1.13 |
| non-SMC condensin II complex, subunit D3 (Ncapd3) | -1.68 | 0.01 | -1.21 |
| exostoses (multiple) 1 (Ext1) | -1.70 | 0.00 | -1.19 |
| thyrotroph embryonic factor (Tef), transcript variant 1 | -1.70 | 0.04 | -1.18 |
| coiled-coil domain containing 120 (Ccdc120) | -1.70 | 0.00 | -1.22 |
| CTD (carboxy-terminal domain, RNA polymerase II, polypeptide A) small phosphatase-like (Ctdspl) | -1.70 | 0.02 | -1.24 |
| MAF1 homolog (Maf1) | -1.72 | 0.00 | -1.13 |
| solute carrier family 25, member 28 (Slc25a28) | -1.73 | 0.02 | -1.18 |
| unconventional SNARE in the ER 1 homolog (Use1), transcript variant 1 | -1.76 | 0.00 | -1.15 |
| transketolase (Tkt) | -1.76 | 0.05 | -1.18 |
| ATPase, Na+/K+ transporting, alpha 1 polypeptide (Atp1a1) | -1.77 | 0.00 | -1.09 |
| nephronophthisis 4 (juvenile) homolog (human) (Nphp4) | -1.78 | 0.02 | -1.25 |
| interleukin enhancer binding factor 3 (Ilf3) | -1.78 | 0.02 | -1.18 |
| casein kinase 1, epsilon (Csnk1e) | -1.78 | 0.00 | -1.18 |
| cholecystokinin B receptor (Cckbr) | -1.78 | 0.02 | -1.15 |
| RIKEN cDNA 1700019D03 gene (1700019D03Rik) | -1.79 | 0.01 | -1.25 |
| transmembrane protein 110 (Tmem110) | -1.80 | 0.00 | -1.21 |
| ribosomal protein S8 (Rps8) | -1.80 | 0.00 | -1.11 |
| microtubule-associated protein 2 (Mtap2), transcript variant 2 | -1.81 | 0.00 | -1.11 |
| gamma-aminobutyric acid (GABA(A)) receptor-associated protein-like 1 (Gabarapl1) | -1.82 | 0.03 | -1.14 |
| non-SMC element 2 homolog (MMS21, S. cerevisiae) (Nsmce2) | -1.83 | 0.00 | -1.22 |
| TRAF type zinc finger domain containing 1 (Trafd1) | -1.83 | 0.00 | -1.22 |
| ubiquitin specific peptidase 52 (Usp52) | -1.84 | 0.02 | -1.27 |
| RNA binding motif protein 5 (Rbm5) | -1.84 | 0.00 | -1.16 |
| megakaryocyte-associated tyrosine kinase (Matk) | -1.86 | 0.01 | -1.20 |
| dapper homolog 2, antagonist of beta-catenin (xenopus) (Dact2) | -1.87 | 0.03 | -1.23 |
| cold inducible RNA binding protein (Cirbp) | -1.87 | 0.03 | -1.25 |
| G protein-coupled receptor associated sorting protein 1 (Gprasp1), transcript variant 3 | -1.88 | 0.01 | -1.10 |
| transmembrane protein 201 (Tmem201), transcript variant 1 | -1.90 | 0.03 | -1.24 |
| expressed sequence AI316807 (AI316807) | -1.90 | 0.00 | -1.19 |
| ilvB (bacterial acetolactate synthase)-like (Ilvbl) | -1.92 | 0.00 | -1.27 |
| leukocyte tyrosine kinase (Ltk), transcript variant 2 | -1.92 | 0.00 | -1.27 |
| resistance to inhibitors of cholinesterase 3 homolog (Ric3), transcript variant 1 | -1.92 | 0.01 | -1.24 |
| complexin 1 (Cplx1) | -1.95 | 0.00 | -1.10 |
| protein tyrosine phosphatase, receptor type, D (Ptprd), transcript variant a | -1.95 | 0.00 | -1.16 |
| upstream binding protein 1 (Ubp1) | -1.99 | 0.01 | -1.22 |
| peroxisome proliferative activated receptor, gamma, coactivator 1 alpha (Ppargc1a) | -2.01 | 0.01 | -1.26 |
| eukaryotic translation elongation factor 1 alpha 2 (Eef1a2) | -2.01 | 0.01 | -1.12 |
| RAS protein-specific guanine nucleotide-releasing factor 1 (Rasgrf1), transcript variant 1 | -2.02 | 0.01 | -1.11 |
| dihydropyrimidinase-like 4 (Dpysl4) | -2.05 | 0.03 | -1.21 |
| Rho GTPase-activating protein (Grit) | -2.05 | 0.00 | -1.17 |
| trinucleotide repeat containing 6a (Tnrc6a) | -2.07 | 0.01 | -1.23 |
| src homology 2 domain-containing transforming protein D (Shd) | -2.07 | 0.00 | -1.24 |
| synaptic vesicle glycoprotein 2 a (Sv2a) | -2.07 | 0.00 | -1.15 |
| golgi apparatus protein 1 (Glg1) | -2.08 | 0.00 | -1.15 |
| ankyrin 1, erythroid (Ank1) | -2.08 | 0.04 | -1.27 |
| rhomboid domain containing 2 (Rhbdd2) | -2.10 | 0.02 | -1.31 |
| thymosin, beta 10 (Tmsb10) | -2.11 | 0.02 | -1.22 |
| visinin-like 1 (Vsnl1) | -2.12 | 0.00 | -1.13 |
| mahogunin, ring finger 1 (Mgrn1) | -2.13 | 0.00 | -1.17 |
| leucine rich repeat transmembrane neuronal 1 (Lrrtm1) | -2.14 | 0.00 | -1.30 |
| prolylcarboxypeptidase (angiotensinase C) (Prcp) | -2.18 | 0.00 | -1.31 |
| guanylate cyclase 1, soluble, alpha 3 (Gucy1a3) | -2.20 | 0.04 | -1.20 |
| potassium voltage gated channel, Shaw-related subfamily, member 4 (Kcnc4) | -2.21 | 0.02 | -1.30 |
| nuclear receptor binding protein 2 (Nrbp2) | -2.22 | 0.01 | -1.23 |
| syntaxin binding protein 2 (Stxbp2) | -2.22 | 0.00 | -1.28 |
| cadherin 8 (Cdh8), transcript variant 1 | -2.28 | 0.02 | -1.34 |
| microtubule associated serine/threonine kinase 1 (Mast1) | -2.36 | 0.00 | -1.33 |
| cholinergic receptor, muscarinic 3, cardiac (Chrm3) | -2.40 | 0.00 | -1.33 |
| cell adhesion molecule with homology to L1CAM (Chl1) | -2.44 | 0.02 | -1.35 |
| ATPase inhibitory factor 1 (Atpif1), nuclear gene encoding mitochondrial protein | -2.45 | 0.00 | -1.16 |
| myocyte enhancer factor 2C (Mef2c) | -2.46 | 0.01 | -1.18 |
| cadherin, EGF LAG seven-pass G-type receptor 3 (Celsr3) | -2.48 | 0.04 | -1.28 |
| sestrin 1 (Sesn1) | -2.56 | 0.00 | -1.29 |
| neurexin I (Nrxn1) | -2.58 | 0.03 | -1.25 |
| peptidase domain containing associated with muscle regeneration 1 (Pamr1) | -2.59 | 0.05 | -1.36 |
| trinucleotide repeat containing 6C (Tnrc6c) | -2.64 | 0.01 | -1.32 |
| ovostatin homolog (Ovos) | -2.74 | 0.00 | -1.36 |
| unc-13 homolog B (Unc13b) | -2.77 | 0.02 | -1.30 |
| LUC7-like 3 (Luc7l3) | -2.78 | 0.00 | -1.27 |
| A kinase (PRKA) anchor protein 8-like (Akap8l) | -2.79 | 0.00 | -1.31 |
| abhydrolase domain containing 14b (Abhd14b) | -2.79 | 0.00 | -1.34 |
| gene model 1821, (NCBI) (Gm1821) on chromosome 14 | -2.83 | 0.02 | -1.17 |
| ubiquitin specific peptidase 2 (Usp2), transcript variant 2 | -2.85 | 0.00 | -1.32 |
| amnionless (Amn) | -2.90 | 0.03 | -1.40 |
| teashirt zinc finger family member 3 (Tshz3) | -2.92 | 0.00 | -1.33 |
| Kruppel-like factor 5 (Klf5) | -2.92 | 0.03 | -1.41 |
| doublecortin (Dcx), transcript variant 4 | -3.00 | 0.00 | -1.42 |
| odd Oz/ten-m homolog 4 (Odz4) | -3.03 | 0.00 | -1.34 |
| special AT-rich sequence binding protein 1 (Satb1) | -3.08 | 0.01 | -1.30 |
| family with sequence similarity 5, member C (Fam5c) | -3.10 | 0.04 | -1.40 |
| paraneoplastic antigen MA3 (Pnma3) | -3.10 | 0.00 | -1.40 |
| netrin G1 (Ntng1) | -3.18 | 0.01 | -1.43 |
| small nucleolar RNA host gene 11 (Snhg11) | -3.44 | 0.05 | -1.28 |
| zinc finger, matrin type 4 (Zmat4) | -3.46 | 0.00 | -1.48 |
| hydroxy-delta-5-steroid dehydrogenase, 3 beta- and steroid delta-isomerase 2 (Hsd3b2) | -3.50 | 0.02 | -1.30 |
| predicted gene, EG665378 (EG665378) | -3.52 | 0.02 | -1.43 |
| nuclear factor I/X (Nfix), transcript variant 2 | -3.77 | 0.00 | -1.42 |
| DNA segment, human D4S114 (D0H4S114) | -4.77 | 0.03 | -1.64 |
